# Orexin A Differentially Influences the Extinction Retention of Recent and Remote Fear Memory

**DOI:** 10.3389/fnins.2018.00295

**Published:** 2018-05-03

**Authors:** Le Shi, Wenhao Chen, Jiahui Deng, Sijing Chen, Ying Han, Muhammad Z. Khan, Jiajia Liu, Jianyu Que, Yanping Bao, Lin Lu, Jie Shi

**Affiliations:** ^1^Department of Pharmacology, School of Basic Medical Sciences, Peking University Health Science Center, Beijing, China; ^2^National Institute on Drug Dependence and Beijing Key Laboratory of Drug Dependence Research, Peking University, Beijing, China; ^3^Peking University Sixth Hospital, Peking University Institute of Mental Health, Key Laboratory of Mental Health, Ministry of Health, National Clinical Research Center for Mental Disorders, Peking University Sixth Hospital, Peking University, Beijing, China; ^4^Peking-Tsinghua Center for Life Sciences and PKU-IDG/McGovern Institute for Brain Research, Peking University, Beijing, China

**Keywords:** recent fear memory, remote fear memory, extinction, orexin A, fear return

## Abstract

Recently the role of the orexin system in the learning and memory, especially orexin A, which could enhance fear memory through regulating the activity of amygdala, has drawn considerable attention. However, the relationship between orexin A and extinction memory remains unclear. To investigate the effect of orexin A on extinction memory in humans, we recruited 43 male subjects and divided them into a recent group and remote group. After acquiring Pavlovian fear conditioning, individuals in recent group experienced fear extinction 24 h after acquisition, and remote group underwent extinction 2 weeks later. Meanwhile, plasma orexin A levels before extinction were measured by enzyme-linked immunosorbent assay. Both groups received memory test 24 h after fear extinction. The results showed that both recent and remote groups successfully acquired fear conditioning and had spontaneous recovery at test. In particular, the correlational analysis indicated that orexin A levels before extinction were negatively associated with fear responses during test only in recent group, but not in remote group. Moreover, individuals with high orexin A levels still kept low fear responses after extinction in recent group by subgroup analyses. The results suggest that orexin A could influence the retention of recent fear memory extinction, without affecting remote fear extinction. These findings remind us the orexin system can be a potential treatment target for fear-related disorders, and the mechanisms of recent and remote fear extinction may be different.

## Introduction

Fear memory could help us to adapt for survival, since it guides individuals to avoid dangerous situations. However, excessive fear could lead to fear-related disorders, such as posttraumatic stress disorder (PTSD) (Izquierdo et al., 2016). The prevalence of PTSD is as high as 12.5% in primary care patients and 25% in soldiers (Spottswood et al., 2017). Currently, in clinic the first-line treatment for fear-related disorders is exposure therapy, which just temporally inhibits the original fear memory, and the extinguished fear often returns when re-exposure to the traumatic events (reinstatement) and re-encountering the trauma associated cues out of the extinction environment (renewal), and after time passes by (spontaneous recovery) (Rescorla and Heth, 1975; Bouton and King, 1983; Quirk, 2002). This reminds us that investigating the mechanisms of fear-related disorders and the influence factors of exposure therapy is essential for the prevention and treatment of fear-related disorders.

According to Pavlovian fear conditioning theory, neutral conditioned stimuli (CS) combined with aversive unconditioned stimulus (US) could elicit fear responses (Domjan, 2005). Once fear memory has been encoded, it goes into consolidation process. There are two kinds of interactive memory consolidation: the short-term cellular/synaptic level (synaptic consolidation) and the long-term brain systems level (systems consolidation) (Dudai, 2012; Kandel et al., 2014). After consolidation, the labile memory becomes stable, and recent and remote fear memory forms. Comparing with recent fear memory (usually forms in several hours), remote fear memory needs days and years to form and has more significant clinical implications (Bergstrom, 2016; Doron and Goshen, 2017). When individuals were continuously exposed to CS in the absence of US, the fear responses would be decreased. This process is called “extinction,” which is the basis of exposure therapy. Extinction does not erase the original fear memory, but forms a new CS− no US extinction memory to compete with fear memory (Milad and Quirk, 2012; Vervliet et al., 2013). Manipulations that enhance extinction are vital for improving the efficacy of exposure therapy.

Recently, the orexin system has attracted growing attention on its role in fear memory. Orexins are secreted by the hypothalamus and have two types, orexin A and orexin B (Sakurai et al., 1998). In contrast to orexin B, which only acts on orexin receptor-2, orexin A could bind with both orexin receptors-1 and 2, and thus exerts more physiological effects (Sakurai et al., 1998). Animal research has shown that the prepro-orexin mRNA levels increased after an episode of footshocks, and were positively associated with the immobilizing time (Chen et al., 2014). In addition, the non-selective dual orexin receptor antagonists could reduce fear response and disrupt the generalized avoidance behavior after electric shocks (Viviani et al., 2015). In humans, the relationship between orexins and fear and anxiety has also been partially detected. A previous study has shown that narcolepsy patients, the good model to study the effect of orexins on fear memory due to their congenital deficiency of orexin (Peyron et al., 2000), had no activation of amygdala and failed to acquire fear memory during aversive conditioning (Ponz et al., 2010). Moreover, patients with panic disorders often have elevated orexin A levels (Johnson et al., 2010). These findings indicate that the orexin system in amygdala might be a potential target for treating fear-related disorders.

To our knowledge, no study has investigated the relationship between orexin A and extinction memory in humans. In this study, we explored the role of orexin A in the fear memory retrieval, extinction learning and retention under laboratory settings, to provide some evidence in the area of the orexin system and fear memory for clinical therapy.

## Materials and methods

### Participants

Forty-three young healthy native Han Chinese men were recruited through posters and online advertisements. Subjects included in this study should be between the age of 18 and 30 years with a body mass index (BMI) of 18.0–30.0 kg/m^2^ and have generally good health as determined by a physician. Those with a history of mental and neurological disorders, sleep disturbances, physical impairment, cardiovascular disease, metabolic syndrome, substances abuse (alcohol, drugs, smoking, and medications) and other diseases were excluded. Moreover, individuals who took medications, including chemical drugs and traditional Chinese medicine, were also excluded. This study was approved by the *Institutional Review Board of Peking University Sixth Hospital*. Each participant signed a consent form. Once completed the experiment, they would be paid USD$50 each for their participation.

### Experimental design

Subjects were asked to arrive at the lab at 8:00 a.m. after finishing their breakfast on the first day. Then they read the consent form and signed it. The participants who were willing to enroll the study were scheduled for a screening interview, during which the data of disease history and demographic information were collected. Subjects reported their age, education levels and exercise frequencies, and BMI was calculated according to height and weight. Since mood state and general cognitive function might affect the behavioral performance (McDonald et al., 2004; Bauer, 2015; Izquierdo et al., 2016), we used the Self-rating Depression Scale (SDS), the Self-rating Anxiety Scale (SAS) and the Montreal Cognitive Assessment (MoCA) to evaluate the baseline depression, anxiety and cognitive function, respectively.

**Figure 1:**
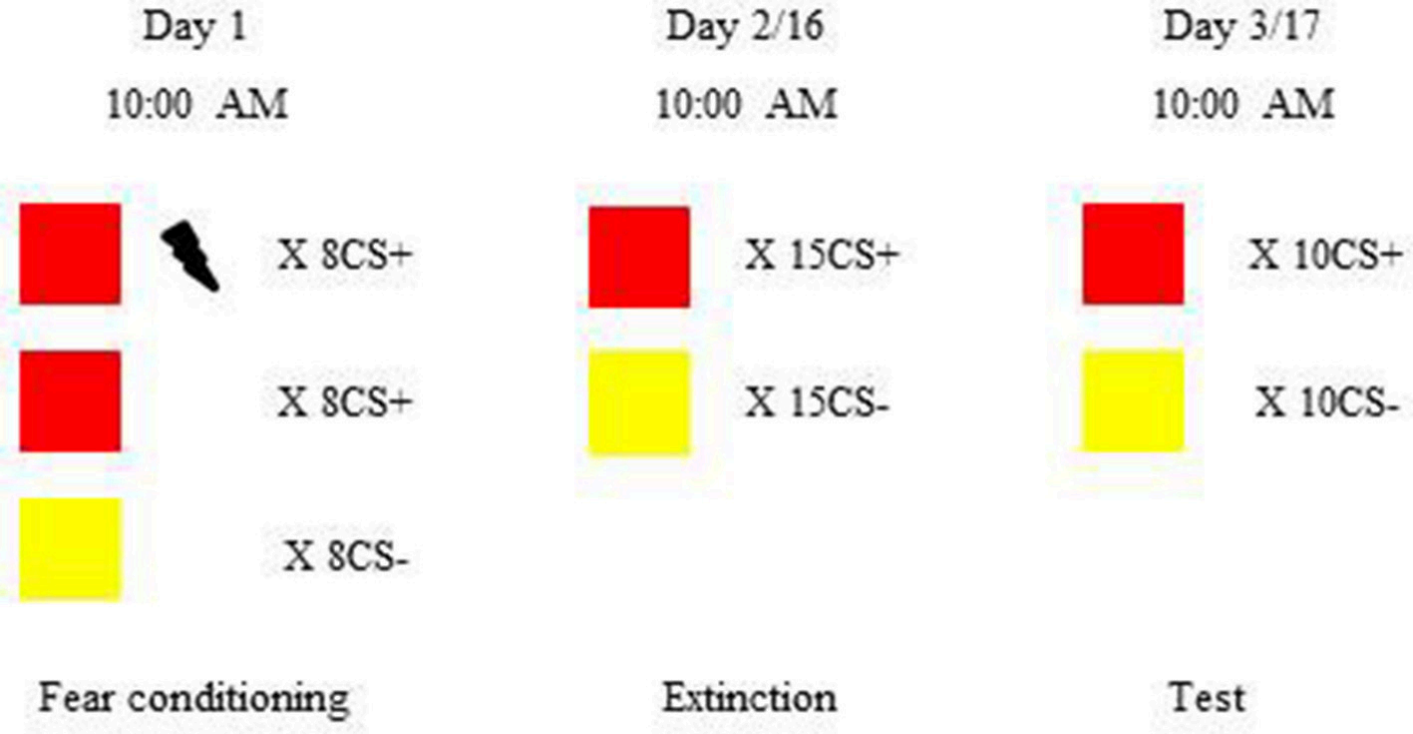
Procedure and timeline of the experiment. Subjects were trained to learn visual cued fear conditioning and then were divided into the recent group and remote group. The recent group underwent extinction 24 h after fear acquisition. The remote group underwent extinction 2 weeks after learning. Twenty-four hours after extinction, both the recent and remote groups were tested for fear expression. In visual cued fear conditioning, red and yellow colored squares were chosen as conditioned stimuli (CS). The unconditioned stimulus (US) was an electric shock to the right inner wrist. During fear acquisition, all of the subjects learned the associations between the CS and US. There were eight reinforced CS+, eight non-reinforced CS+, and eight CS−. Extinction consisted of 15 trials, each with one non-reinforced CS+ and one CS−. In the fear memory test, 10 CS+ without the US and 10 CS− were presented. To avoid the effects of circadian rhythms, sleep, and feeding on orexin A levels, all of the sesions occurred at 10:00 a.m.

After completing the questionnaires, all subjects underwent fear conditioning, and then were divided into two groups randomly: recent group (*n* = 21) and remote group (*n* = 22). Recent group went through extinction 24 h after fear learning, while remote group experienced extinction 2 weeks later. Memory test was performed 24 h after extinction (see Figure 1). In order to exclude the effects of circadian rhythms, sleep and feeding on the orexins levels (Huang et al., 2011; Patton and Mistlberger, 2013), all participants were asked to wake up at 7:00 a.m. and eat breakfast between 7:30 to 8:00 a.m., and went through fear acquisition, extinction learning and memory test at 10:00 a.m.

### Fear conditioning paradigm

Visual cued fear conditioning paradigm (see Figure 1) was used in this study, and the procedure was based on our previous studies (Liu et al., 2014; Ai et al., 2015; He et al., 2015). For fear conditioning, red and yellow colored squares were chosen as CS and mild electric shock to the right inner wrist was treated as US and delivered by a constant-current STM200 stimulator (BIOPAC Systems, Goleta, CA, USA). Before the experiment, the electric shock level was individually determined based on the criterion “very uncomfortable, but not painful.”

During fear acquisition, one of the CS would be paired with US under a partial reinforcement schedule (50% reinforced) and be considered as the CS+, and another CS (CS−) was never combined with US. To adjust for the influence of color itself on fear acquisition, two different orders of presentations were used to counterbalance designations of squares as the CS+ or CS−. There were eight non-reinforced presentations of each CS, intermixed with eight additional reinforced CS+ presentations in the fear acquisition. To avoid the influence of electric shock itself on fear responses, we only included the non-reinforced CS+ when calculating the fear expression during fear learning.

Extinction consisted of 15 trials, each containing one non-reinforced CS+ and one CS−. The average differential fear responses to the first three trials were considered as the retrieval of acquired fear memory, and the extinction score was calculated by the average difference of fear responses to the last three CS+ and the last three CS−. Moreover, to detect the effect of orexin A on extinction process, we defined extinction slope as the average differential fear responses to the first three presentations of each CS minus the average differential fear responses to the last three presentations of each CS.

Twenty-four hours after extinction, subjects underwent memory test. There were 10 non-reinforced trials each including one CS+ and one CS− during test. The test score was the mean differential fear response to the first three CS. Other squares were designed to re-extinction in order to diminish the fear.

In all stages, the CS were presented on the computer using E-Prime 2.0 professional software (Psychology Software Tools, Inc.; http://www.pstnet.com), and subjects were asked to focus on the relationship between CS presentation and occurrence of electric shock. The durations of CS were 4 s, and the shocks were administered for 500 ms, which coterminated with the pictures. Each CS was followed by an interstimulus interval of 8–12 s, during which the participants looked at a fixation point.

### Psychophysiological recording and assessment

The skin conductance response (SCR) was used to reflect the fear response. Two Ag-AgCl electrodes connected to a BIOPAC MP150 system were attached to the second and third fingers of the left hand to measure the SCR. The SCR waveforms were recorded and analyzed using AcqKnowledge software (BIOPAC Systems) and the trough-to-peak difference for each waveform in a 6-s window following stimulus onset was considered as the SCR to the CS. After square-root transforming the original data to normalize the distribution, the differential SCR was obtained by subtracting responses to the CS− from responses to the CS+ in the corresponding trials. At each stage, we calculated the mean differential SCR in the included trials.

### Orexin a measurement

Half an hour before extinction, we collected 10 ml venous blood of each subject using tubes containing 7.5% ethylenediamine tetra-acetic acid tri-potassium salt [EDTA (k3)] anti-coagulant. The blood samples were centrifuged at 3,000 rotations per minute at 4°C for 10 min to obtain plasma and then stored at −80°C until analysis. Plasma orexin A levels were measured using enzyme-linked immunosorbent assay (Phoenix Pharmaceuticals, Belmont, CA, USA; catalog no. EKE-003-30). The intra- and inter-assay coefficients of variation were <10%.

### Statistical analysis

The quantitative data are expressed as mean ± standard error of mean (SEM). We used independent-sample *t*-test to analyze the differences of demographic data, SDS, SAS, MoCA, and shock intensity between recent group and remote group, and χ^2^ test to compare the difference of exercise frequencies between two groups. The Shapiro–Wilk test confirmed that the data had a normal distribution (Table S1). When analyzing the acquisition data, paired-sample *t*-test was used to compare the differences of the mean SCR to CS+ and CS− in recent and remote groups, respectively. Repeated-measures analysis of variance (ANOVA) was used to analyze the group differences of the mean differential SCR in three stages (acquisition, extinction and memory test) with group (recent group and remote group) or subgroup (low orexin A levels and high orexin A levels) as between-subjects factor and stage (acquisition, extinction and test) as within-subjects factor. Significant main effects in the repeated-measures ANOVA were followed by multiple-comparison *post-hoc* tests, and significant interactions were followed by *t*-tests. Pearson correlation coefficients (*r*) were used to evaluate relationships among orexin A levels before extinction and fear memory retrieval, extinction slope and test score (see Fear conditioning paradigm).

## Results

### Demographic data

No significant differences in age, level of education, height, weight, BMI, SDS, SAS, MoCA, or shock intensity were observed between two groups by independent-sample *t*-tests, and the χ^2^ test showed that exercise frequencies were comparable between the two groups (all *p* > 0.05; Table 1). These results indicated that the baseline covariates that might influence orexin A levels and fear memory were comparable between two groups.

**Table 1:** Demographic data and shock intensity of recent and remote groups.

	**Recent (*n* = 21)**	**Remote (*n* = 22)**	**t/χ^2^**	***p***
Age (years)	24.048 ± 0.470	23.818 ± 0.541	0.319	0.752
Education (years)	16.952 ± 0.422	17.227 ± 0.431	−0.455	0.652
Height (cm)	175.238 ± 1.163	173.364 ± 0.929	1.265	0.213
Weight (kg)	70.333 ± 1.511	65.500 ± 2.598	1.589	0.120
BMI	22.903 ± 0.429	21.738 ± 0.778	1.294	0.203
Exercise	18 (85.714%)	19 (86.364%)	0.004	0.951
SDS	37.976 ± 1.197	35.682 ± 1.842	1.034	0.307
SAS	33.929 ± 1.286	35.000 ± 1.605	−0.518	0.607
MoCA	28.000 ± 0.324	27.955 ± 0.275	0.107	0.915
Shock intensity	46.167 ± 2.211	50.773 ± 2.407	−1.406	0.167

*The results are expressed as mean ± SEM. BMI, Body Mass Index; SDS, Self-rating Depression Scale; SAS, Self-rating Anxiety Scale; MoCA, Montreal Cognitive Assessment*.

### Both recent and remote fear memory showed spontaneous recovery after extinction

**Figure 2:**
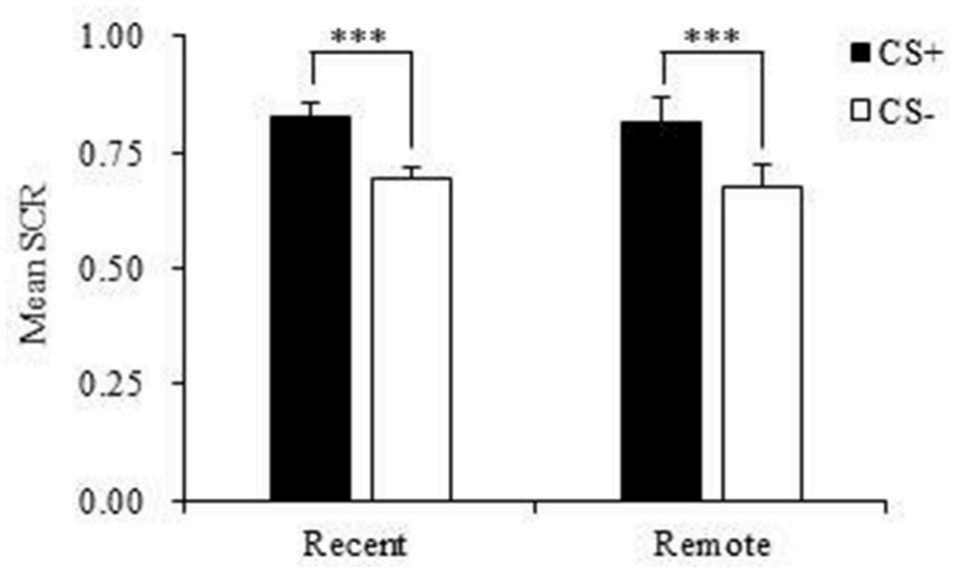
Mean skin conductance response (SCR) in the recent group and remote group during fear acquisition. Both the recent and remote groups had a significantly higher mean SCR to the CS+ compared with the CS−. CS, conditioned stimulus. ***p < 0.001. Error bars represent SEM.

During fear acquisition, we first used paired-sample *t*-tests to evaluate whether the subjects in each group successfully acquired fear memory. The results showed that both the recent group and remote group had a significantly higher fear response to the CS+ compared with the CS− and achieved fear learning (both *p* < 0.001; Figure 2, Table 2).

**Table 2:** Mean skin conductance response (SCR) during fear acquisition in the recent and remote groups.

	**CS+**	**CS−**	***t* (df)**	***p***
Recent (*n* = 21)	0.826 ± 0.029	0.691 ± 0.026	11.678(20)	<0.001
Remote (*n* = 22)	0.816 ± 0.050	0.677 ± 0.045	4.862(21)	<0.001

*The results are expressed as mean ± SEM. CS, conditioned stimulus*.

**Figure 3:**
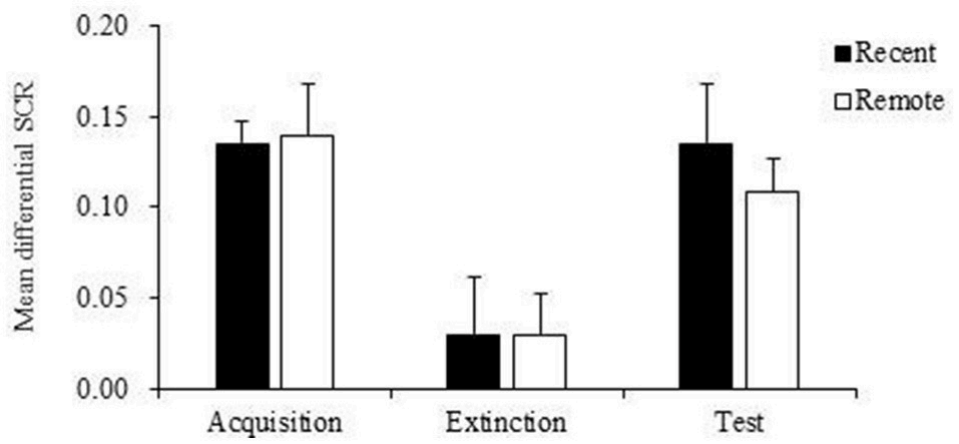
Mean differential skin conductance response (SCR) of recent group and remote group during fear acquisition, extinction and test. The mean differential SCR at extinction were significantly lower than fear expressions in the fear acquisition (*p* < 0.001) and test (*p* < 0.01). Error bars represent SEM.

Then we compared the mean differential SCR differences between the two groups during acquisition, extinction and memory test. The repeated-measures ANOVA indicated no significant main effect of group (*p* = 0.756) and no interaction (*p* = 0.784), suggesting that the recent group and remote group had comparable fear acquisition, extinction, and test (Table 3). However, we found a significant main effect of stage (*p* < 0.001; Table 3). The multiple-comparison *post-hoc* test showed that the mean differential SCR in the last three extinction trials was significantly lower than the SCR in the fear acquisition (*p* < 0.001) and test (*p* < 0.01) sessions. These results indicated that after extinction, both groups formed extinction memory and exhibited lower fear responses, but the new CS− no US memory was not permanent, and fear returned over time (Figure 3).

**Table 3:** Mean differential skin conductance response (SCR) during the fear acquisition, extinction, and test sessions in the recent and remote groups.

	**Acquisition**	**Extinction**	**Test**	**Group/Subgroup**		**Stage**		**Interaction**	
				***F* (*df*)**	***p***	***F* (*df*)**	***p***	***F* (*df*)**	***p***
Overall				0.098 (1, 41)	0.756	12.786 (2, 82)	< 0.001	0.244 (2, 82)	0.784
Recent (*n* = 21)	0.135 ± 0.012	0.030 ± 0.031	0.135 ± 0.033						
Remote (*n* = 22)	0.139 ± 0.029	0.030 ± 0.022	0.109 ± 0.018						
Recent				6.273 (1, 19)	0.022	6.033 (2, 38)	0.005	3.615 (2, 38)	0.037
Low (*n* = 13)	0.137 ± 0.018	0.052 ± 0.041	0.204 ± 0.031						
High (*n* = 8)	0.132 ± 0.008	−0.005 ± 0.045	0.022 ± 0.051						
Remote				0.001 (1, 20)	0.982	6.382 (2, 40)	0.004	0.201 (2, 40)	0.818
Low (*n* = 13)	0.149 ± 0.044	0.025 ± 0.030	0.105 ± 0.024						
High (*n* = 9)	0.125 ± 0.033	0.036 ± 0.035	0.115 ± 0.028						

*The results are expressed as mean ± SEM. CS, conditioned stimulus. Low, low orexin A levels; High, high orexin A levels*.

### Plasma orexin a levels were negatively correlated with the spontaneous recovery of recent fear memory

**Figure 4:**
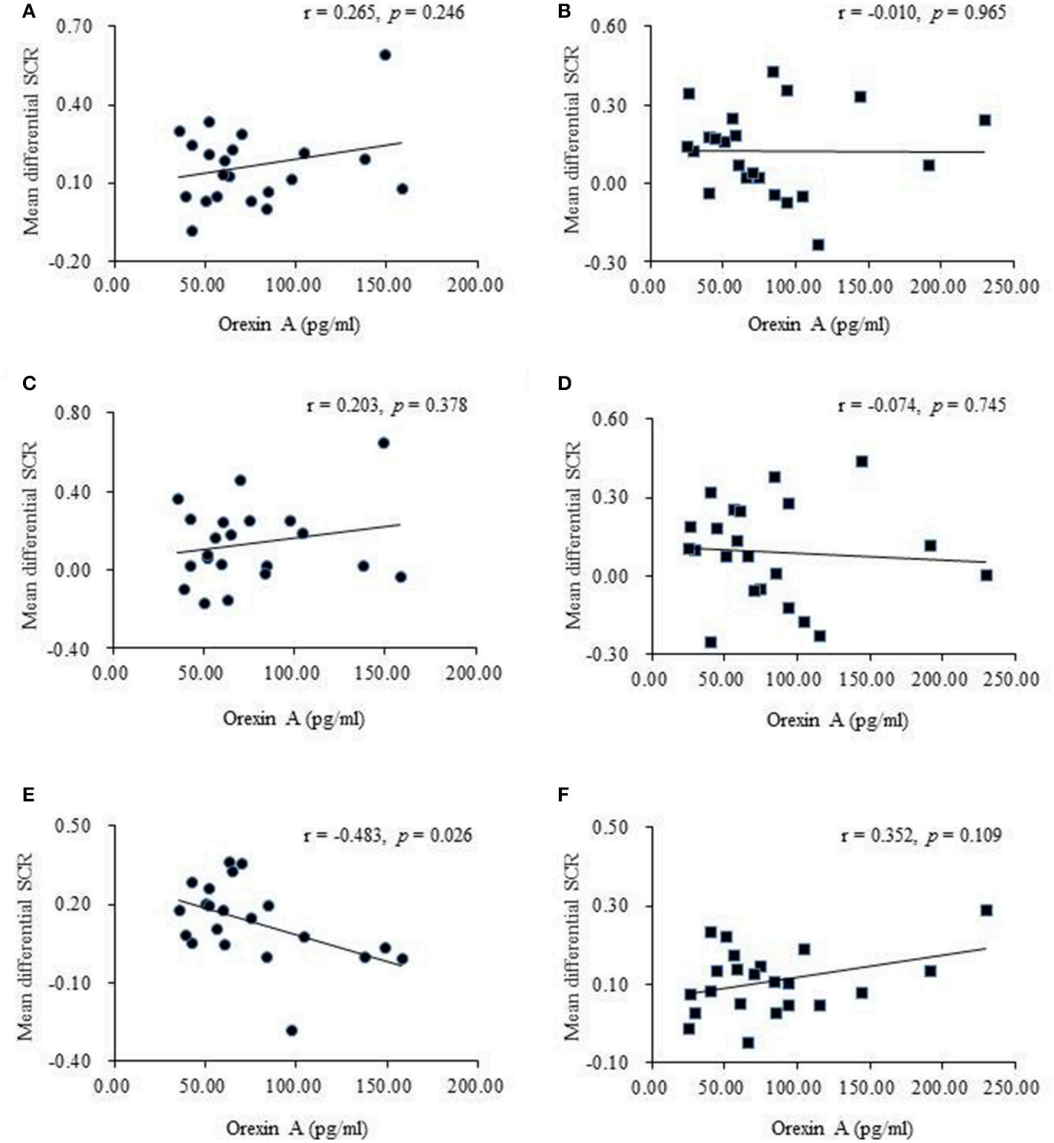
Correlational analyses between plasma orexin A levels and behavioral performances. **(A)** Pearson's correlation between orexin A levels and the mean differential skin conductance response (SCR) in fear memory retrieval in recent group. **(B)** Pearson's correlation between orexin A levels and the mean differential SCR in fear memory retrieval in remote group. The fear memory retrieval was calculated using the first three trials of extinction. **(C)** Pearson's correlation between orexin A levels and the mean differential SCR in extinction slope in recent group. **(D)** Pearson's correlation between orexin A levels and the mean differential SCR in extinction slope in remote group. The extinction slope was calculated by subtracting responses to the last three trials of extinction from responses to the first three trials of extinction. **(E)** Pearson's correlation between orexin A levels and the mean differential SCR at test in recent group. **(F)** Pearson's correlation between orexin A levels and the mean differential SCR at test in remote group. The test score was calculated using the first three trials of test.

In both the recent and remote groups, no significant relationship was found between orexin A levels before extinction and the mean differential SCR in the first three extinction trials or between orexin A levels before extinction and the extinction slope (Figures 4A–D), indicating that orexin A might not be involved in fear memory retrieval or the extinction learning process. However, orexin A might play a significant role in the retention of recent fear memory extinction but not in remote fear memory. The results showed that higher plasma orexin A levels were associated with lower test scores in the recent group (Figure 4E) but not in the remote group (Figure 4F).

### Subjects with high orexin a levels in the recent group exhibited no spontaneous recovery

**Figure 5:**
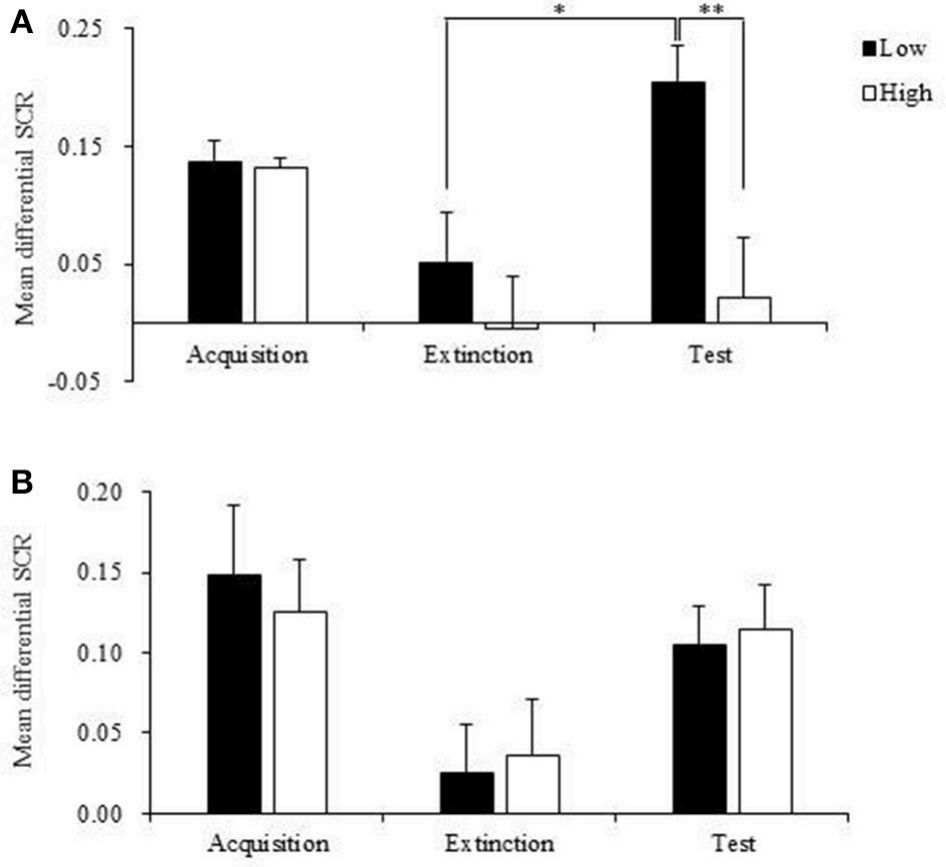
Mean differential skin conductance response (SCR) of low and high orexin A levels subgroups in recent and remote groups during fear acquisition, extinction and test. **(A)** Mean differential SCR of low and high orexin A levels subgroups in recent group during fear acquisition, extinction and test. Low orexin A levels subgroup showed increased fear responses at test compared to that during extinction and the fear expressions at test in high orexin A levels subgroup. *p < 0.05, **p < 0.01. **(B)** Mean differential SCR of low and high orexin A levels subgroups in remote group during fear acquisition, extinction and test. Compared to extinction, the fear responses significantly increased at test in both low and high orexin A levels subgroups (*p* < 0.01). Error bars represent SEM.

Subjects in recent group and remote group each were divided into two subgroups according to the plasma orexin A levels. The individuals with higher than mean orexin A levels were regarded as the high subgroup, and those with orexin A levels lower than mean value were treated as the low subgroup. The repeated-measures ANOVA showed that in the recent group, there were significant main effects of stage (*p* = 0.005) and subgroup (*p* = 0.022) on fear responses and a stage × subgroup interaction (*p* = 0.037; Table 3). The *post-hoc t*-test revealed that individuals with relatively high orexin A levels exhibited no spontaneous recovery in the test session (*p* = 0.963; Figure 5A), and fear expression in the subgroup with low orexin A significantly increased in the test session compared with the extinction session (*p* = 0.017; Figure 5A). In the remote group, no significant main effect of subgroup (*p* = 0.982) and no stage × subgroup interaction (*p* = 0.818) were observed, but a significant main effect of stage was found (*p* = 0.004), indicating that orexin A did not influence remote fear memory extinction or extinction recall (Figure 5B, Table 3). These results confirmed that high orexin A concentration might be a protective factor for recent fear memory extinction.

## Discussion

In this study, we found that extinction could only temporarily inhibit recent and remote fear memory, and the baseline orexin A level may be related to the extinction retention of recent fear memory, but not remote fear memory. These findings indicate that first, extinction is not stable, and identifying the factors that might enhance extinction is necessary; second, orexin A might be a potential target for promoting recent fear memory extinction; third, the extinction processes of recent and remote fear memory share different molecular mechanisms, which should be explored separately.

Extinction-based exposure therapy has been widely used for the treatment of fear-related disorders. However, 19–62% patients relapsed after receiving exposure therapy (Vervliet et al., 2013). Our study under the laboratory settings proved that both recent and remote fear memory could not be erased by extinction as the fear responses increased 24 h after extinction. Therefore, it is important to explore mechanisms of extinction to promote the development of new treatment. The dsregulation of orexin A levels has recently been reported in individuals with fear-related disorders, including high orexin A levels in panic disorder and low orexin A levels in PTSD (Johnson et al., 2010; Strawn et al., 2010). Moreover, narcolepsy patients with inherent orexin deficiency failed to acquire fear (Ponz et al., 2010). These clinical findings indicate that the orexin system, especially orexin A, is a key regulator in the development of fear-related disorders.

The present study used a Pavlovian fear conditioning paradigm and found that orexin A levels before extinction were not associated with recent or remote fear memory retrieval or extinction learning processes. Nevertheless, we found a negative association between plasma orexin A levels and recent fear memory expression after extinction. High orexin A levels might help subjects avoid the spontaneous recovery of recent fear memory after extinction. These results indicate that orexin A may enhance the consolidation and retention of recent fear memory extinction.

Some studies have explored the role of orexin A in memory consolidation. Orexin A binds orexin-1 and -2 receptors to exert considerable physiological functions (Sakurai et al., 1998). However, the distributions of orexin-1 and -2 receptors in fear-related brain areas are distinct, and they play differential roles in memory consolidation (Flores et al., 2015). Administration of an orexin-1 receptor antagonist impaired both hippocampus-dependent spatial memory and amygdala-dependent emotional memory (Akbari et al., 2007; Ardeshiri et al., 2017). Orexin A treatment reversed the disruption of memory under conditions of orexin deficiency (Zhao et al., 2014; Mavanji et al., 2017). However, orexin-2 receptor activation had little and even opposite effects on memory consolidation (Sears et al., 2013; Soya et al., 2013; Ardeshiri et al., 2017). These findings suggest that orexin A mainly binds orexin-1 receptors to exert its memory-enhancing effect.

To date, only a few studies have directly investigated the association between orexin system and fear memory extinction. Two animal studies found that orexin-1 receptor antagonist facilitates cue- and context-dependent fear extinction by activating basolateral amygdala (Flores et al., 2014, 2017). Another rat study showed that high cue-induced freezing during extinction was associated with a greater percentage of activated orexin neurons in the medial hypothalamus during extinction recall, and rats with good extinction learning exhibited less activation of orexin neurons (Sharko et al., 2017). The disparate results of the present study and previous studies appear to be attributable to different species and methods. Some evidence suggests that the interaction between orexin A and orexin-1 receptors could increase neuronal firing in the CA1 area of the hippocampus and promote neurogenesis in the dentate gyrus (Zhao et al., 2014; Chen et al., 2017). Orexin A infusion enhanced hippocampal long-term potentiation through the co-activation of plasticity-related kinases (Wayner et al., 2004; Selbach et al., 2010; Lu et al., 2016), which is critical for memory consolidation (Schafe et al., 2001). The hippocampus is a key node in the consolidation and retention of recent fear memory extinction (Myskiw et al., 2014; Fullana et al., 2018). We propose that the enhancing effect of orexin A on recent fear extinction memory mainly depends on hippocampal neurogenesis and synaptic plasticity.

We also found that orexin A only influenced recent fear memory extinction, without affecting remote fear memory extinction. As previously mentioned, hippocampus is an important region through which orexin A enhances memory by promoting neurogenesis and synaptic plasticity. According to the memory consolidation theory, recent memory firstly stores in the hippocampus and then transfers to neocortex for long-term storage later (Dudai, 2012). Orexin A scarcely had an effect on neocortex when regulating emotional memory (Flores et al., 2014). Additionally, the application of orexin A to cortical neuron cultures suppressed *N*-methyl-D-aspartate receptor expression (Raoof et al., 2015), the downstream signaling of which has been shown to be involved in extinguishing remote fear memory (Corcoran et al., 2013). These findings may explain the differential effects of orexin A on recent and remote fear memory extinction. However, the mechanisms of orexin A's differential regulation of recent and remote fear memory are still unknown, and further studies of the relationship between orexin A and fear extinction are needed.

There are several limitations in this study. First, to exclude the influence of menstrual cycle, we only recruited male subjects, and the findings might not be applied in female subjects. Second, Pavlovian fear conditioning was performed in a laboratory setting, thus the generalizability of the results to clinical patients should be used in caution. Third, we only measured the association between plasma orexin A levels and memory. Causal relationships are unclear, and plasma orexin A levels may not necessarily reflect levels in the brain. Fourth, the sample in our study was relatively small. In the future, we should test the effect of exogenous orexin A supplement on exposure therapy in patients with fear-related disorders with a large sample.

In conclusion, our results suggest orexin A may be associated with recent fear memory extinction retention, but not remote fear extinction retention, and subjects with high orexin A levels have decreased recent fear memory expressions after extinction. Furthermore, these findings suggest that though recent and remote fear memory returns after extinction, the mechanisms between them might be different, and orexin A only involved in the retention of recent fear memory extinction, without affecting remote fear memory extinction. Future studies should further explore the role of orexin A in the process of recent and remote fear memory extinction and extend the findings to clinical population.

## Ethics statement

This study was carried out in accordance with the recommendations of ethical standards of the Institutional Review Board of Peking University Sixth Hospital. The protocol was approved by the Institutional Review Board of Peking University Sixth Hospital. All subjects gave written informed consent in accordance with the Declaration of Helsinki.

## Author contributions

LS, JS, and LL: Designed the study; LS, JD, JQ, and SC: Performed the experiments; LS, WC, SC, JL, and YB: Analyzed data and wrote the paper; MK, YH, JS, and LL: Commented on and edited the manuscript.

### Conflict of interest statement

The authors declare that the research was conducted in the absence of any commercial or financial relationships that could be construed as a potential conflict of interest.
