# Supplementary material for: Orexin A Differentially Influences the Extinction Retention of Recent and Remote Fear Memory
Source: Front Neurosci. 2018 May 3;12:295. doi: 10.3389/fnins.2018.00295 (PMC5943634; doi:10.3389/fnins.2018.00295)
Supplement: Supplementary file 1 [file Table1.DOC]

**Table S1. The normality of the data using Shapiro-Wilk methods.**

|  | **Statistic** | **df** | ***p*** |
| --- | --- | --- | --- |
| **Acquisition** |  |  |  |
| **Recent group*** | **0.963** | **21** | **0.588** |
| **CS+** | **0.964** | **21** | **0.603** |
| **CS-** | **0.962** | **21** | **0.549** |
| **Low subgroup** | **0.914** | **13** | **0.210** |
| **High subgroup** | **0.988** | **8** | **0.991** |
| **Remote group*** | **0.929** | **22** | **0.116** |
| **CS+** | **0.972** | **22** | **0.753** |
| **CS-** | **0.974** | **22** | **0.795** |
| **Low subgroup** | **0.878** | **13** | **0.066** |
| **High subgroup** | **0.914** | **9** | **0.346** |
| **Extinction** |  |  |  |
| **Recent group*** | **0.977** | **21** | **0.881** |
| **Low subgroup** | **0.944** | **13** | **0.511** |
| **High subgroup** | **0.966** | **8** | **0.863** |
| **Remote group*** | **0.985** | **22** | **0.971** |
| **Low subgroup** | **0.974** | **13** | **0.935** |
| **High subgroup** | **0.965** | **9** | **0.848** |
| **Test** |  |  |  |
| **Recent group*** | **0.940** | **21** | **0.218** |
| **Low subgroup** | **0.938** | **13** | **0.429** |
| **High subgroup** | **0.871** | **8** | **0.155** |
| **Remote group*** | **0.986** | **22** | **0.983** |
| **Low subgroup** | **0.973** | **13** | **0.926** |
| **High subgroup** | **0.888** | **9** | **0.191** |

*** indicates the mean differential skin conductance response in the group. CS, Conditioned Stimuli.**
